# Application of a conical transparent cap for the retrieval of extraluminal foreign bodies

**DOI:** 10.1016/j.igie.2025.07.009

**Published:** 2025-08-05

**Authors:** Jia-Huan Liu, Bin-Yang Luo, Zhu Wang

**Affiliations:** Department of Gastroenterology and Hepatology, West China Hospital, Sichuan University, Chengdu, China

Foreign body (FB) ingestion involving the esophagus constitutes a common medical emergency. We report a novel application of a conical transparent cap, traditionally used for endoscopic retrograde appendicitis therapy, in the successful retrieval of an extraluminal FB.

A 65-year-old man presented with an 8-day history of dysphagia after swallowing a duck bone. Contrast-enhanced computed tomography with intravenous contrast only (no oral contrast administered) revealed a 2-cm linear, high-density FB situated extraluminally at the upper esophagus, without evidence of pneumomediastinum (Fig. 1A). Gastroscopy revealed 2 mucosal defects at the esophageal entrance but no visible FB (Fig. 1B). The initial procedural priority involved localization of the FB and achievement of adequate exposure. The endoscope distal cap served to stabilize the endoscopic position, thereby enabling significant expansion of both the visual field and operative workspace. Correlating with computed tomography imaging, the bone was localized to the right side. Initial attempts using a standard transparent cap (D-201-11804; Olympus, Tokyo, Japan) to retrieve the bone through the right defect were unsuccessful, as scope stabilization at the narrow esophageal entrance was challenging, and the tangential viewing angle hindered precise manipulation. To address these limitations, another cap (DH-28GR; Fujifilm, Tokyo, Japan), known for its suitability in endoscopic dissecting, was used. Its tapered tip and silicone grip ring improve endoscope stability, visualization, and instrument control in narrow or angulated regions. Nevertheless, retrieval attempts remained unsuccessful. Finally, a conical transparent cap (20222060845; Diyi Medical, Henan, China) was used (Fig. 1C). Its tapered end facilitated partial dilation of the defect and exposure of the bone, while also providing enhanced stability (Fig. 1D). This allowed the forceps to penetrate deeper into the esophageal wall, successfully retrieving the bone (Fig. 1E, Video 1, available online at www.igiejournal.org). Given infected surrounding tissues, we opted for nasogastric tube placement instead of immediate perforation closure after FB removal. Postprocedural management included a 7-day course of broad-spectrum antibiotics (piperacillin-tazobactam) combined with gastric acid suppression therapy (omeprazole). The patient was discharged and transferred to a secondary care facility to finish antimicrobial therapy.

**Figure 1:**
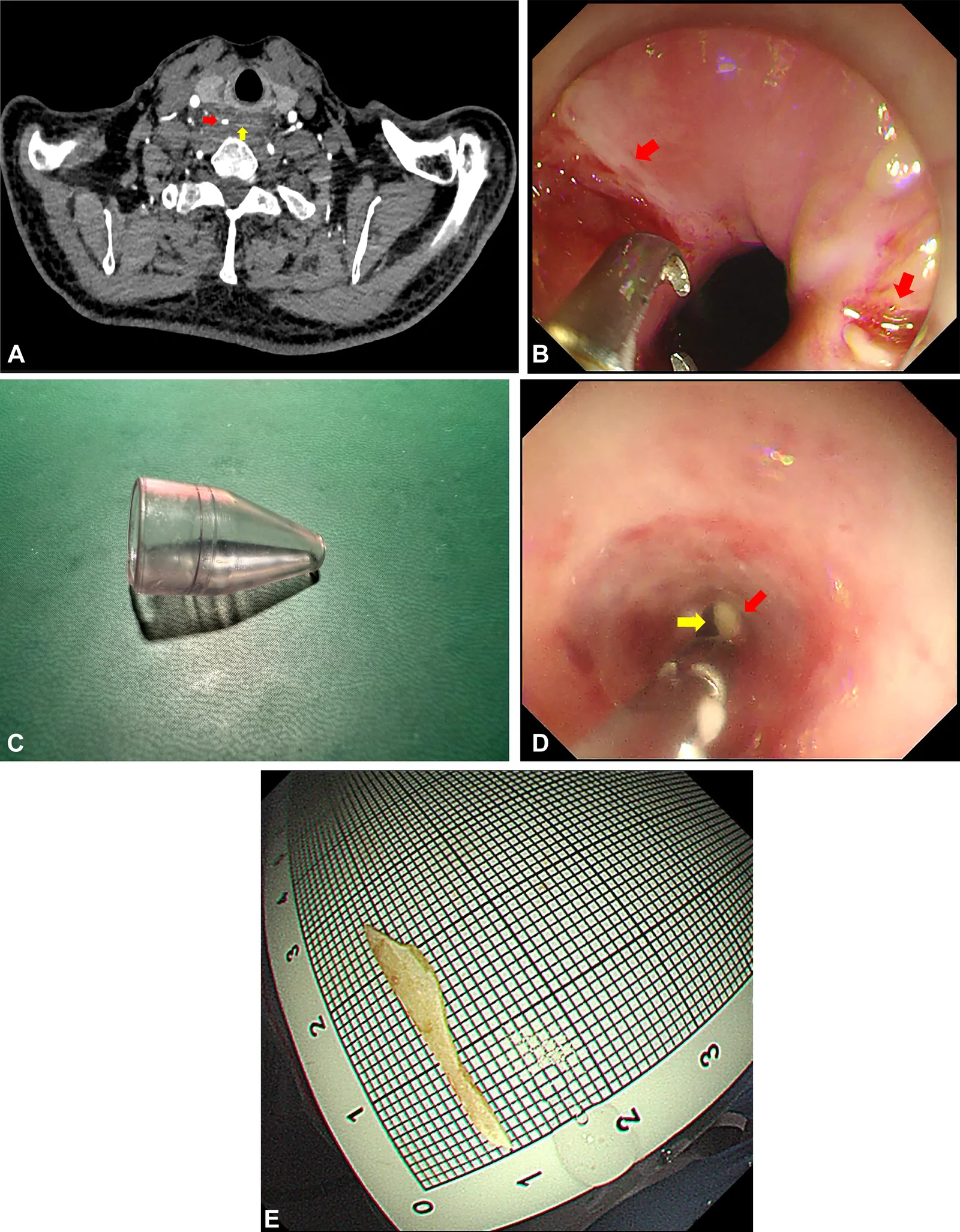
**A,** Computed tomography scan showing a 2-cm linear, high-density object consistent with a foreign body (indicated by the *red arrow*) located extraluminally at the upper esophagus. The *yellow arrow* marks the esophageal lumen. **B,** Gastroscopic view of the esophageal entrance showing 2 mucosal defects (*red arrows*), suggesting possible perforation, although no visible foreign body was detected intraluminally. **C,** Photograph of the conical transparent distal cap used during endoscopy. **D,** Endoscopic image during retrieval demonstrating the utility of the conical transparent cap in stabilizing the field of view and facilitating exposure. The *red arrow* indicates the identified foreign body, whereas the *yellow arrow* denotes the perforation site. **E,** Photograph of the retrieved foreign body, identified as a duck bone.

The selection of an appropriate distal attachment is a pivotal consideration in endoscopic FB retrieval. In this case, the conical transparent cap proved instrumental by stabilizing the scope, dilating the narrow defect, and providing sufficient exposure to facilitate successful removal. This novel application highlights the potential of conical transparent caps in managing challenging cases of extraluminal FBs, underscoring their clinical utility and versatility.

## Patient consent

Informed consent was obtained from the patient for the publication.

## Disclosure

All authors disclosed no financial relationships. This study was funded by the National Key Research and Development Program of China (2022YFC3602101).

## Supplementary data

Video 1 Endoscopic retrieval of an extraluminal duck bone with different types of caps. A conical transparent cap provided enhanced stability, partial dilation of the defect, and exposure of the foreign body, successfully retrieving the bone.

